# Supplementary material for: Comparative analysis of the gut microbiota of sand fly vectors of zoonotic visceral leishmaniasis (ZVL) in Iran; host-environment interplay shapes diversity
Source: PLoS Negl Trop Dis. 2022 Jul 19;16(7):e0010609. doi: 10.1371/journal.pntd.0010609 (PMC9337680; doi:10.1371/journal.pntd.0010609)
Supplement: S1 Table — P: pathogen, NP: non-pathogen, M: northwest, B: northeast, S: southwest. (DOCX) [file pntd.0010609.s001.docx]

**Table S1.** Taxonomic, types of gram stain, characters, and the number of operational taxonomic units (OTUs) in sand fly female guts revealed by NGS. P: pathogen, NP: non-pathogen, M: northwest, B: northeast, S: southwest.

| Species | Gram  **+** **/** - | Character | No. of OTUs in | | | | | | |
| --- | --- | --- | --- | --- | --- | --- | --- | --- | --- |
|  |  |  | *P. kandelakii-*M | *P. kandelakii-*B | *P. major-*B | *P. perfiliewi-* M | *P. alexandri-* B | *P. alexandri-*S | Total |
| *Acinetobacter*  *lwoffii* | Neg | Pathogen | 0 | 38 | 0 | 0 | 0 | 0 | 38 |
| *Acinetobacter baumannii* | Neg | Pathogen | 0 | 0 | 0 | 0 | 0 | 7 | 7 |
| *Acinetobacter calcoaceticus* | Neg | Pathogen | 0 | 0 | 0 | 0 | 0 | 44 | 44 |
| *Actinokineospora diospyrosa* | + | Pathogen | 0 | 0 | 0 | 0 | 0 | 25 | 25 |
| *Actinomadura*  *vinacea* | + | Pathogen  (low) | 0 | 0 | 0 | 0 | 0 | 55 | 55 |
| *Actinophytocola algeriensis* | + | Pathogen | 0 | 0 | 0 | 0 | 0 | 40 | 40 |
| *Aeromonas* sp. | Neg | Pathogen | 69 | 0 | 0 | 34 | 26 | 0 | 129 |
| *Aggregatibacter aphrophilus* | Neg | Pathogen | 0 | 0 | 0 | 0 | 0 | 2 | 2 |
| *Allokutzneria albata* | + | Pathogen | 0 | 0 | 0 | 0 | 0 | 61 | 61 |
| *Arthrobacter*  *agilis* | + | Pathogen | 55 | 24 | 0 | 67 | 0 | 0 | 146 |
| *Bacillus*  *licheniformis* | + | Pathogen | 36 | 173 | 0 | 0 | 0 | 0 | 209 |
| *Bacillus*  *subtilis* | + | Non-pathogen | 23 | 0 | 68 | 43 | 42 | 78 | 254 |
| *Bacillus soli* | + | Non-pathogen | 0 | 0 | 0 | 0 | 0 | 29 | 29 |
| *Bacillus sp.* | + | Non-pathogen | 0 | 0 | 48 | 0 | 0 | 0 | 48 |
| *Blastococcus massiliensis* | + | Pathogen | 0 | 0 | 0 | 62 | 0 | 0 | 62 |
| *Cellulosimicrobium cellulans* | + | Pathogen | 0 | 0 | 0 | 0 | 0 | 36 | 36 |
| *Clostridiales sp* | Both | Pathogen  opportunistic | 0 | 35 | 0 | 0 | 0 | 0 | 35 |
| *Clostridium asparagiforme* | + | Pathogen | 0 | 0 | 0 | 34 | 0 | 0 | 34 |
| *Clostridium perfringens* | + | Pathogen | 0 | 45 | 0 | 64 | 0 | 0 | 109 |
| *Comamonas*  *aquatica* | Neg | Non-pathogen | 54 | 0 | 0 | 0 | 0 | 0 | 54 |
| *Comamonas*  *kerstersii* | Neg | Non-pathogen | 33 | 0 | 0 | 0 | 0 | 0 | 33 |
| *Cupriavidus*  *gilardii* | Neg | Non-pathogen | 41 | 71 | 0 | 0 | 0 | 0 | 112 |
| *Desemzia*  *incerta* | + | Pathogen | 31 | 0 | 0 | 51 | 0 | 21 | 103 |
| *Enterobacter*  *cloacae* | Neg | Pathogen | 24 | 0 | 0 | 38 | 80 | 97 | 239 |
| *Enterobacter aerogenes* | Neg | Pathogen | 0 | 0 | 0 | 29 | 0 | 0 | 29 |
| *Enterobacter asburiae* | Neg | Pathogen | 0 | 0 | 0 | 20 | 0 | 0 | 20 |
| *Firmicutes sp* | + | Non-pathogen | 0 | 0 | 0 | 0 | 0 | 23 | 23 |
| *Frondihabitans australicus* | + | Pathogen | 56 | 0 | 0 | 0 | 0 | 0 | 56 |
| *Geobacillus stearothermophilus* | + | Non-pathogen | 0 | 0 | 71 | 0 | 0 | 0 | 71 |
| *Georgenia*  *halophila* | + | Pathogen | 0 | 70 | 0 | 0 | 0 | 0 | 72 |
| *Glutamicibacter nicotianae* | + | Pathogen | 65 | 0 | 0 | 0 | 0 | 0 | 65 |
| *Helicobacter*  *ganmani* | Neg | Pathogen | 0 | 46 | 0 | 0 | 59 | 91 | 196 |
| *Jeotgalicoccus coquinae* | + | Pathogen | 0 | 0 | 55 | 0 | 0 | 0 | 55 |
| *Kaistia*  *geumhonensis* | Neg | Pathogen  opportunistic | 0 | 0 | 0 | 32 | 0 | 0 | 32 |
| *Klebsiella*  *variicola* | Neg | Pathogen | 0 | 0 | 0 | 43 | 0 | 0 | 43 |
| *Klebsiella pneumoniae* | Neg | Pathogen | 0 | 0 | 0 | 15 | 0 | 0 | 15 |
| *Kocuria*  *palustris* | + | Non-pathogen | 66 | 106 | 72 | 42 | 0 | 0 | 286 |
| *Kocuria*  *turfanensis* | + | Non-pathogen | 46 | 0 | 61 | 0 | 0 | 0 | 107 |
| *Lactobacillus acidophilus* | + | Non-pathogen | 19 | 86 | 0 | 0 | 0 | 0 | 105 |
| *Lactobacillus delbrueckii* | + | Non-pathogen | 9 | 0 | 0 | 0 | 0 | 0 | 9 |
| *Lactobacillus dextrinicus* | + | Non-pathogen | 43 | 0 | 0 | 0 | 0 | 0 | 43 |
| *Lactobacillus fermentum* | + | Non-pathogen | 0 | 0 | 0 | 0 | 0 | 9 | 9 |
| *Lactobacillus salivarius* | + | Non-pathogen | 75 | 0 | 0 | 77 | 0 | 0 | 152 |
| *Lysinibacillus macroides* | + | Pathogen  (Insect) | 12 | 0 | 0 | 41 | 0 | 0 | 53 |
| *Lysinibacillus sphaericus* | + | Pathogen  (Insect) | 199 | 84 | 0 | 82 | 0 | 0 | 365 |
| *Massilia timonae* | Neg | Non-pathogen | 0 | 0 | 66 | 0 | 0 | 0 | 66 |
| *Morganella*  *morganii* | Neg | Pathogen | 26 | 0 | 0 | 0 | 0 | 0 | 26 |
| *Neisseria*  *meningitidis* | Neg | Pathogen | 0 | 0 | 0 | 0 | 0 | 63 | 63 |
| *Nesterenkonia*  *lutea* | + | Pathogen | 0 | 34 | 0 | 0 | 0 | 0 | 34 |
| *Nocardia cyriacigeorgica* | + | Pathogen | 30 | 0 | 0 | 0 | 0 | 0 | 30 |
| *Nocardioides deserti* | + | Pathogen | 22 | 0 | 0 | 0 | 0 | 0 | 22 |
| *Nocardioides kribbensis* | + | Pathogen | 69 | 0 | 0 | 0 | 0 | 0 | 69 |
| *Paraclostridium bifermentans* | + | Pathogen | 0 | 0 | 0 | 98 | 0 | 20 | 118 |
| *Paracoccus*  *chinensis* | Neg | Pathogen  opportunistic | 0 | 23 | 0 | 0 | 0 | 0 | 23 |
| *Pedobacter glucosidilyticus* | neg | Non-pathogen | 0 | 16 | 0 | 0 | 0 | 0 | 16 |
| *Planococcus donghaensis* | + | Non-pathogen | 0 | 0 | 0 | 0 | 0 | 17 | 17 |
| *Pontibacter*  *humi* | neg | Pathogen | 0 | 0 | 0 | 88 | 0 | 0 | 88 |
| *Promicromonospora aerolata* | + | Pathogen | 0 | 0 | 0 | 0 | 0 | 40 | 40 |
| *Pseudomonas*  *putida* | Neg | Pathogen | 0 | 0 | 0 | 0 | 0 | 56 | 56 |
| *Pseudomonas*  *stutzeri* | Neg | Pathogen | 32 | 0 | 0 | 0 | 0 | 0 | 32 |
| *Pseudomonas aeruginosa* | Neg | Pathogen | 78 | 0 | 106 | 191 | 0 | 47 | 422 |
| *Pseudomonas mendocina* | Neg | Pathogen | 0 | 0 | 0 | 0 | 0 | 15 | 15 |
| *Pseudonocardia ailaonensis* | + | Pathogen  (opportunistic) | 0 | 0 | 0 | 0 | 0 | 28 | 28 |
| *Psychrobacter alimentarius* | Neg | Pathogen | 0 | 0 | 0 | 0 | 0 | 27 | 27 |
| *Rathayibacter festucae* | + | Pathogen | 0 | 0 | 0 | 0 | 101 | 0 | 101 |
| *Rhizobiales sp.* | Neg | Pathogen | 0 | 0 | 0 | 0 | 33 | 0 | 33 |
| *Sphingobacterium bambusae* | neg | Pathogen | 0 | 0 | 0 | 39 | 0 | 0 | 39 |
| *Staphylococcus aureus* | + | Pathogen | 0 | 24 | 0 | 0 | 0 | 0 | 24 |
| *Streptococcus constellatus* | + | Non-pathogen | 0 | 0 | 137 | 85 | 0 | 0 | 222 |
| *Veillonella*  *parvula* | Neg | Pathogen | 0 | 0 | 0 | 0 | 25 | 0 | 25 |
| *Virgibacillus carmonensis* | + | Pathogen | 0 | 0 | 0 | 0 | 0 | 24 | 24 |
| *Wolbachia sp* | Neg | Non-pathogen | 0 | 0 | 0 | 46 | 0 | 25609 | 25609 |
| **Total (%)** | +/- 41/30  (57.7) | **Pathogen / non-pathogen (%)** | 804/409  (66.3) | 596/279  (68.1) | 161/523  (23.5) | 1028/247  (80.6) | 324/42  (88.5) | 799/156  (83.6)* | 5368  (69.15)* |
