# Supplementary material for: Comparative analysis of the gut microbiota of sand fly vectors of zoonotic visceral leishmaniasis (ZVL) in Iran; host-environment interplay shapes diversity
Source: PLoS Negl Trop Dis. 2022 Jul 19;16(7):e0010609. doi: 10.1371/journal.pntd.0010609 (PMC9337680; doi:10.1371/journal.pntd.0010609)
Supplement: Attachment — Submitted filename: Responses to all review comments.docx [file pntd.0010609.s002.docx]

**Responses to all review comments,**

Your manuscript has undergone careful revision by two reviews, and we are pleased to inform you that both have suggested that your manuscript be accepted with minor revisions and modification to one figure to make it more comprehensible.

**Response:**

Thanks for your positive response and accepting our manuscript. The figure has been modified accordingly and new version has been provided.

Additionally please note that “All data is presented in the manuscript” is not an appropriate data availability statement for a microbiome profiling experiment. 16S sequences should be submitted to an appropriate repository (Genbank SRA) and accessions listed in the data availability section.

**Response:**

The sequence data have been submitted to Genbank SRA and their accession numbers have been provided and addressed in the text.

Reviewer's Responses to Questions

<b>Key Review Criteria Required for Acceptance?</b></br></br>

As you describe the new analyses required for acceptance, please consider the following:</br></br>

<b>Methods</b></br></br>

-Are the objectives of the study clearly articulated with a clear testable hypothesis stated?</br>

-Is the study design appropriate to address the stated objectives?</br>

-Is the population clearly described and appropriate for the hypothesis being tested?</br>

-Is the sample size sufficient to ensure adequate power to address the hypothesis being tested?</br>

-Were correct statistical analysis used to support conclusions?</br>

-Are there concerns about ethical or regulatory requirements being met?</br></br>

Reviewer #1: yes

Reviewer #2: The experimental design employed in this study was adequate to study objectives, the comparison of the microbiota composition of four sand flies species from three different geographic locations in Iran, using 16S RNA gene sequencing technique. My critical observation is about the number of specimens analyzed, been just 48 sand flies female samples. A higher number of insects could produce more robust results.

**Response:**

We accept the reviewer comment and definitely more sand flies could produce more robust results. However, we collected few specimens for some species and hence we tried to use almost similar number of specimens per species to have a more reliable comparison.

--------------------

<b>Results</b></br></br>

-Does the analysis presented match the analysis plan?</br>

-Are the results clearly and completely presented?</br>

-Are the figures (Tables, Images) of sufficient quality for clarity?</br></br>

Reviewer #1: figures need some updates

Reviewer #2: The results are clearly described, and the figures are clear and easy to understand, except for the figure 3- I, the Venn diagram. I consider this figure confused with many numbers inside and outside the diagram. This visual pollution makes it difficult the result understanding showed in this figure. I suggest redoing this figure.

**Response:**

Most of figures have been redone.

--------------------

<b>Conclusions</b></br></br>

-Are the conclusions supported by the data presented?</br>

-Are the limitations of analysis clearly described?</br>

-Do the authors discuss how these data can be helpful to advance our understanding of the topic under study?</br>

-Is public health relevance addressed?</br></br>

Reviewer #1: yes

Reviewer #2: The authors conclusions are adequate to results obtained, except to suggestion to use the Asaia bacteria in paratransgeneis approaches. Since it was only found in one species. Bacteria with greater distribution, present in several species, as seen with Bacillus subtilis, present in all species studied, present a greater potential for use in paratransgenic approaches.

**Response**:

The referee is quite right and Asaia sp. Has been found in only one species. However, to nominate a bacterium for paratransgenesis, other characteristics including being non-pathogenic, malleability, trans-stadial and transovarial transmission are also important for nomination. Because this bacterium has these good characteristics for paratransgenesis and previously have been used successfully to develop paratransgenic mosquitoes we have mentioned it in the conclusion.

--------------------

<b>Editorial and Data Presentation Modifications?</b></br><br/>

Use this section for editorial suggestions as well as relatively minor modifications of existing data that would enhance clarity. If the only modifications needed are minor and/or editorial, you may wish to recommend “Minor Revision” or “Accept”.

Reviewer #1: (No Response)

Reviewer #2: I suggest minor changes to the article. First change the Venn diagram figure, so that it is more understandable. Second, to improve the argument to suggest that despite having only been identified in a single species, the authors suggest that Asaia would be a good candidate for paratransgenic modifications.

**Response:**

Thanks for the comment, I would like to see the referee suggestion about the title. The Venn diagram figure has been modified. As it was explained above, to nominate a bacterium for paratransgenesis, characteristics like being non-pathogenic, malleability, trans-stadial and transovarial transmission are also important for nomination. Because this bacterium has got these good characteristics and previously have been used successfully to develop paratransgenic mosquitoes we have mentioned it in the conclusion.

--------------------

<b>Summary and General Comments</b></br></br>

Use this section to provide overall comments, discuss strengths/weaknesses of the study, novelty, significance, general execution and scholarship. You may also include additional comments for the author, including concerns about dual publication, research ethics, or publication ethics. If requesting major revision, please articulate the new experiments that are needed.

Reviewer #1: The paper “Comparative analysis of the gut microbiota of sand fly vectors of zoonotic visceral leishmaniasis (ZVL) in Iran; host-environment interplay shapes diversity” by Prof Mohammad Ali Oshaghi and colleagues is a well-written and welcomed contribution to the field. I only have a few comments and those mainly revolve around the figures and tables that should be made clearer.

Lines 89-91: what does “economical” mean in this context?

**Response:** Because NGS can identify most bacteria (aerobic and an-aerobic, symbiont and non-symbiont, culturable and non-culturable) at the same time it could be economical. However, to avoid confusion we have replaced “economical” with “more wide-ranging “.

Line 133: not clear what you are dissecting.

**Response:** We meant dissecting “guts”. It was corrected accordingly.

Line 183: 160550 -> 160,500

**Response**: It was corrected accordingly.

Line 259: be more precise in what is “much much more”, a number or so

**Response**: It was corrected accordingly and numbers have been shown.

Fig. 2.: change so that the same phylum has the same color in all diagrams, now e.g. Cyanobacteria has six different colors, which makes it difficult to compare

**Response**: It was corrected accordingly, and same color has been used for same pylum.

Fig. 4.: put genera in alphabetical order

**Response**: It was corrected accordingly.

Fig. 6.: not clear what red and blue means, what is yellow?

**Response**: The colors have been deleted.

Fig. 7.: explain purple, pink, yellow, and other colors and also the different geometrical forms

**Response**: The colors and geometrical forms have been identified.

Table 1.: check space between genus and species, here and in the rest of the manuscript.

**Response**: It was corrected accordingly.

Table 2.: singular and plural for taxonomic levels should be adjusted

**Response**: It was corrected accordingly.

Table 3.: put as supplementary data and put species in alphabetical order

**Response**: The table was put as a supplementary data and the species ordered alphabetically.

Reviewer #2: In this descriptive article, the authors compare the gut bacterial microbiota composition from sand flies of four different species and three geographic locations in Iran. Using 16 S high throughput DNA sequencing technique the authors observed that the bacterial microbiota composition was related to both, sand fly specie and the environment. Sympatric species present different bacterial species in their microbiota. An interesting observation was the relationship between the gut microbiota diversity and the sand fly vectorial capacities, where species presenting more diverse bacterial microbiota composition were the species with higher Leishmania transmission abilities. The identification of several pathogenic bacteria for human and animals in the sand flies guts also suggested a potential role sand flies in the transmission of the bacteria pathogens. Unfortunately in this work alterations of gut sand flies microbiota due to seasonal variations were not

studied. Seasonal alterations could be related to a increase or decrease in pathogens transmission. The acknowledgment about the relationship among the vector, microbiota and pathogens can be used to develop mechanisms to control or eradicate the dissemination of several vector borne diseases. The authors suggest the use of Bacillus subtilis, Enterobacter cloacae and Asaia, as candidates for a paratransgenesis approach to the fight against Leishmaniasis. But from these three putative candidates, the only one found in all four sand flies especies studyed was B. subtilis. Commensals present on vectors species are better candidates for paratransgenesis approaches, than others identified in only one specie as was Asaia in this work.

**Response**:

Thanks for the positive comments and views of respectful reviewer. The referee is right, and we did not study the impact of season on microbiota. We have acknowledged this fact in the discussion.

The reason we have nominated Asia sp for paratransgenesis has been explained above.

--------------------

PLOS authors have the option to publish the peer review history of their article (<a href="<https://journals.plos.org/plosntds/s/editorial-and-peer-review-process#loc-peer-review-history>" target="_blank">what does this mean?</a>). If published, this will include your full peer review and any attached files.<br><br>

If you choose “no”, your identity will remain anonymous but your review may still be made public.<br><br>

<b>Do you want your identity to be public for this peer review?</b> For information about this choice, including consent withdrawal, please see our <a href="<https://www.plos.org/privacy-policy>" target="_blank">Privacy Policy</a>.

Reviewer #1: No

Reviewer #2: No

Figure Files:

While revising your submission, please upload your figure files to the Preflight Analysis and Conversion Engine (PACE) digital diagnostic tool, [https://pacev2.apexcovantage.com](https://pacev2.apexcovantage.com/). PACE helps ensure that figures meet PLOS requirements. To use PACE, you must first register as a user. Then, login and navigate to the UPLOAD tab, where you will find detailed instructions on how to use the tool. If you encounter any issues or have any questions when using PACE, please email us at [figures@plos.org](mailto:figures@plos.org).

Data Requirements:

Please note that, as a condition of publication, PLOS' data policy requires that you make available all data used to draw the conclusions outlined in your manuscript. Data must be deposited in an appropriate repository, included within the body of the manuscript, or uploaded as supporting information. This includes all numerical values that were used to generate graphs, histograms etc.. For an example see here: <http://www.plosbiology.org/article/info%3Adoi%2F10.1371%2Fjournal.pbio.1001908#s5>.

Reproducibility:

To enhance the reproducibility of your results, we recommend that you deposit your laboratory protocols in protocols.io, where a protocol can be assigned its own identifier (DOI) such that it can be cited independently in the future. Additionally, PLOS ONE offers an option to publish peer-reviewed clinical study protocols. Read more information on sharing protocols at <https://plos.org/protocols?utm_medium=editorial-email&utm_source=authorletters&utm_campaign=protocols>

References

Please review your reference list to ensure that it is complete and correct. If you have cited papers that have been retracted, please include the rationale for doing so in the manuscript text, or remove these references and replace them with relevant current references. Any changes to the reference list should be mentioned in the rebuttal letter that accompanies your revised manuscript. If you need to cite a retracted article, indicate the article's retracted status in the References list and also include a citation and full reference for the retraction notice.

__________________________________________________

In compliance with data protection regulations, you may request that we remove your personal registration details at any time. (Use the following URL: <https://www.editorialmanager.com/pntd/login.asp?a=r>). Please contact the publication office if you have any questions.
